# Grain Growth Behavior of 0.95(Na_0.5_Bi_0.5_)TiO_3_–0.05BaTiO_3_ Controlled by Grain Shape and Second Phase

**DOI:** 10.3390/ma13061344

**Published:** 2020-03-16

**Authors:** Sang-Chae Jeon, John G. Fisher, Suk-Joong L. Kang, Kyoung-Seok Moon

**Affiliations:** 1 School of Materials Science and Engineering, Changwon National University, 20 Changwondaehak-ro, Uichang-gu, Changwon, Gyeongsangnam 51140, Korea; scjeon@changwon.ac.kr; 2 School of Materials Science and Engineering, Chonnam National University, 77 Yongbong-ro, Buk-gu, Gwangju 61186, Korea; johnfisher@jnu.ac.kr; 3 Department of Materials Science and Engineering, Korea Advanced Institute of Science and Technology, 291 Daehak-ro, Yuseong-gu, Daejeon 34141, Korea; sjkang@kaist.ac.kr; 4 School of Materials Science and Engineering, Gyeongsang National University, 501 Jinjudaero, Jinju, Gyeongnam 52828, Korea

**Keywords:** grain growth, grain shape, liquid phase, sintering, microstructure, NBT-BT

## Abstract

The grain growth behavior of 0.95(Na_0.5_Bi_0.5_)TiO_3_ –0.05BaTiO_3_ (mole fraction, NBT–5BT) grains was investigated with excess Bi_2_O_3_ addition. The powder compacts of NBT–5BT were sintered at 1200 °C for various sintering times and with various amounts of Bi_2_O_3_ (0.1, 1.5, 4.0 and 10.0 mol%). When Bi_2_O_3_ was added to round-edged cubic NBT–5BT, the grain shape changed to a more faceted cube and the amount of liquid phase increased during sintering. A more faceted cubic grain shape indicates an increase in the critical driving force for appreciable growth of grains. However, obvious abnormal grain growth did not appear in any of the NBT–5BT samples with excess Bi_2_O_3_. The amount of liquid phase increased as the amount of Bi_2_O_3_ increased. Therefore, the rate of grain growth could be decreased by the increasing the distance for the diffusion of atoms. These observations allowed us to conclude that the growth of Bi_2_O_3_-excess NBT–5BT grains is governed by the growth of facet planes via the two-dimensional nucleation grain growth mechanism during changing grain shape and amount of liquid.

## 1. Introduction

The physical properties of ceramic materials are closely related to the microstructure via grain growth and densification, which depends on the structure, composition, shape of grains, and interfacial structure. Physical and chemical properties of materials can be dramatically improved by the development of microstructure via control of grain growth behavior with changing physicochemical properties of the interfaces such as surfaces, liquid/solid interfaces, and grain boundaries [1,2,3,4,5,6]. For example, Kinoshita and Yamaji found that the dielectric constant as a function of temperature in BaTiO_3_ is dependent on grain size [7]. S. Huo *et al*. found that grain size influenced the ferroelectric and piezoelectric properties of (K_0.5_Na_0.5_)NbO_3_ [8]. Recently, studies on microstructural development have reported that interface motion is closely related to the interface structure associated with step free energy, critical driving force for growth, and grain shape [2,3,9,10,11,12,13,14,15,16,17,18,19,20,21].

Usually, to enhance microstructural development such as suppression of grain growth and enhancement of densification, many researchers have studied the effect of additive elements. These additive elements can segregate at grain boundaries and form a secondary solid or a liquid phase. Grain boundary segregation and formation of a secondary solid phase could cause suppression of grain growth by the solute drag and Zener effects [22,23]. A liquid phase can change diffusion parameters such as length of diffusion and rate of movement [23]. In addition, the additives can change the interface structure and energy. However, the effect of additives is not clear for each additive and each system. Kang et al. proposed with experimental and simulation results that the grain growth behavior can be explained by the relationship between the critical driving force and the maximum driving force for growth in the two-dimensional nucleation-controlled growth mechanism [2,3]. Many previous studies [2,11,14,17,18,19,24,25,26,27,28,29,30,31,32] explained and verified the correlation between the equilibrium shape of grains and the critical driving force for growth in which the grain growth behavior was abnormal for faceted grains, but normal for rounded grains. Normal grain growth, observed in systems with round-shaped grains, is caused by diffusion-controlled growth because of an unlimited number of nucleation sites at the rough interface, leading to an absence of a critical driving force for growth. For an atomically ordered interface with faceting, the migration rate of the interface is nonlinear with respect to the driving force, so a critical driving force for growth can exist. Therefore, abnormal grains can be observed in systems with faceted grains because grains with driving force below the critical driving force cannot grow and grains with higher driving force than the critical driving force can grow dramatically [19]. In our previous study, 0.95(Na_0.5_Bi_0.5_)TiO_3_–0.05BaTiO_3_ (NBT–5BT) had a round-edged cubic shape and the grain growth behavior was initially similar to normal grain growth but abnormal grains could be observed as sintering time increased [18]. The abnormal grain growth behavior was obviously increased when TiO_2_ was added because the critical driving force increases [19]. This NBT–5BT system is a candidate as a lead-free piezoelectric material [33,34].

The purpose of the present study was to provide experimental support for the effect of Bi_2_O_3_ addition on grain growth behavior in NBT–5BT. Specifically, the relationship between grain shape and grain growth behavior in the NBT–5BT system was investigated with different additions of Bi_2_O_3_. The grain shape and amount of liquid phase systematically varied with the addition of Bi_2_O_3_. In addition, the secondary solid phase was formed by excess Bi_2_O_3_. In the NBT–5BT system, microstructural development such as changing grain size and densification is one of the important issues to enhance the electrical properties [35,36,37,38].

## 2. Materials and Methods

The conventional mixed oxide technique was used to prepare the powders. Raw materials are commercial powders of Na_2_CO_3_ (99.5%, Acros Organics, Branchburg, NJ, USA), Bi_2_O_3_ (99.9%, Kojundo Chemical Lab Co., Saitama, Japan), BaCO_3_ (99.98%, Sigma-Aldrich, St. Louis, MO, USA), and TiO_2_ (99.9% Sigma-Aldrich, St. Louis, MO, USA). First, 0.95Na_0.5_Bi_0.5_TiO_3_–0.05BaTiO_3_ powder was prepared. The stoichiometric mixtures of powders were ball-milled in a polypropylene bottle with ethanol and yittria-stabilized zirconia balls for 24 h. The dried slurry was crushed in an agate mortar and passed through a 150 mesh sieve. The dried powder was calcined at 800 °C for 4 h in air. The calcined powder was mixed with 0.1, 1.5, 4.0, and 10.0 mol% Bi_2_O_3_, respectively, and ball-milled again for 24 h. After milling, the slurries were again dried, crushed, and passed through a 150 mesh sieve. Powder compacts of 10 mm diameter and 4 mm thickness were prepared by hand pressing in a stainless steel die and then cold isostatically pressed (CIP) under 200 MPa. The compacts were then sintered at 1200 °C for 10 min, 1 h, 4 h, 12 h, and 48 h on a Pt plate in an alumina crucible with a cover in air. The heating and cooling rate was 4 °C/min.

The final powders and sintered samples were characterized using X-ray diffraction (XRD, X’PERT MPD, Philips, Eindhoven, the Netherlands) with a Cu–Kα radiation source (λ = 0.154056 nm). XRD patterns were analyzed using a program (match! ver. 1.11, manufacturer, Bonn, Germany). The sintered samples were sectioned and polished to a 0.25 μm finish. The polished samples were chemically etched in an 83H_2_O·14HNO_3_·3HF (vol%) solution. The microstructures of the samples were observed using a scanning electron microscope (Model SEM515, Philips, Eindhoven, the Netherlands). Grain size distributions were measured from scanning electron microscopy (SEM) micrographs using an image analysis program (Matrox Inspector 2.1, Matrox Electronic Systems, Ltd., Dorval, QC, Canada). At least 300 grains were measured for each sample and then the two-dimensional size distributions were presented as grain size distribution data.

## 3. Results and Discussion

**Figure 1:**
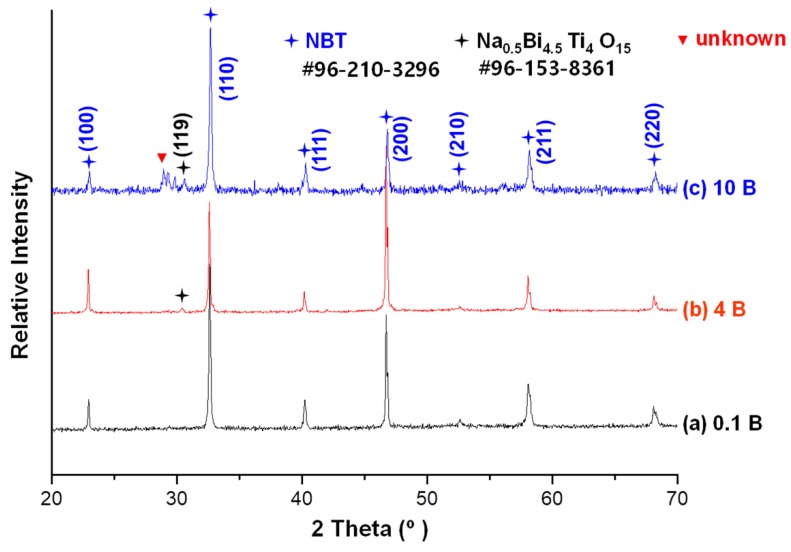
X-ray diffraction (XRD) patterns of Bi_2_O_3_-excess 0.95Na_0.5_Bi_0.5_TiO_3_-0.05BaTiO_3_ (NBT–5BT) sintered at 1200 °C for 4 h in air: (**a**) 0.1 mol% Bi_2_O_3_-excess NBT–5BT; (**b**) 4 mol% Bi_2_O_3_-excess NBT–5BT; (**c**) 10 mol% Bi_2_O_3_-excess NBT–5BT. The star indicates the 2nd phase.

Figure 1 shows X-ray diffraction patterns of the 0.95(Na_0.5_Bi_0.5_)TiO_3_–0.05BaTiO_3_ (NBT–5BT) samples sintered at 1200 °C for 4 h with various amounts of Bi_2_O_3_. It is obvious that single phase NBT–BT appeared below 0.1 mol% of Bi_2_O_3_ (match! ver. 1.11, entry # 96-210-3296). However, in the cases of 4 mol% and 10 mol% Bi_2_O_3_ additions, secondary phase peaks were observed in the XRD patterns. When Bi_2_O_3_ was added to the samples, Bi_2_O_3_ diffused into NBT–BT grains up to the limit of solid solubility. On the other hand, the liquid phase or secondary solid phase was formed by adding excess Bi_2_O_3_. The results presented in Figure 1 indicate that the secondary solid phase can be formed at 4 mol% Bi_2_O_3_ addition and above in this study. The secondary phase is Na_0.5_Bi_4.5_Ti_4_O_15_ (match! ver. 1.11, entry # 96-153-8361), as shown in Figure 1. However, some peaks could not be identified. During sintering, the secondary solid phase could cause suppression of grain growth by the Zener effect [22,23]. However, this result did not inform us of the exact limitation of solubility and the presence of the secondary phase. In the X-ray diffraction analysis, very small amounts of secondary phase could not be detected, so more analysis such as scanning electron microscopy (SEM) and energy dispersive spectroscopy (EDS) was needed.

**Figure 2:**
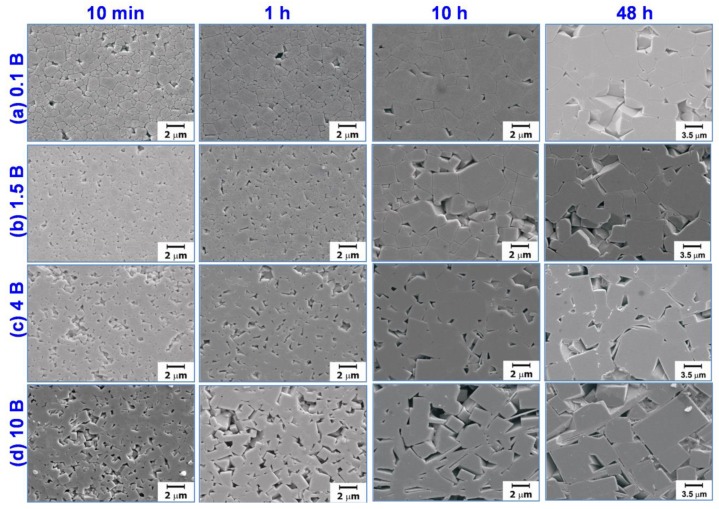
Scanning electron microscopy (SEM) micrographs of (**a**) 0.1 mol%, (**b**) 1.5 mol%, (**c**) 4 mol%, and (**d**) 10 mol% Bi_2_O_3_-excess 0.95(Na_0.5_Bi_0.5_)TiO_3_-0.05BaTiO_3_ (NBT–5BT) samples sintered at 1200 °C for various sintering times.

**Figure 3:**
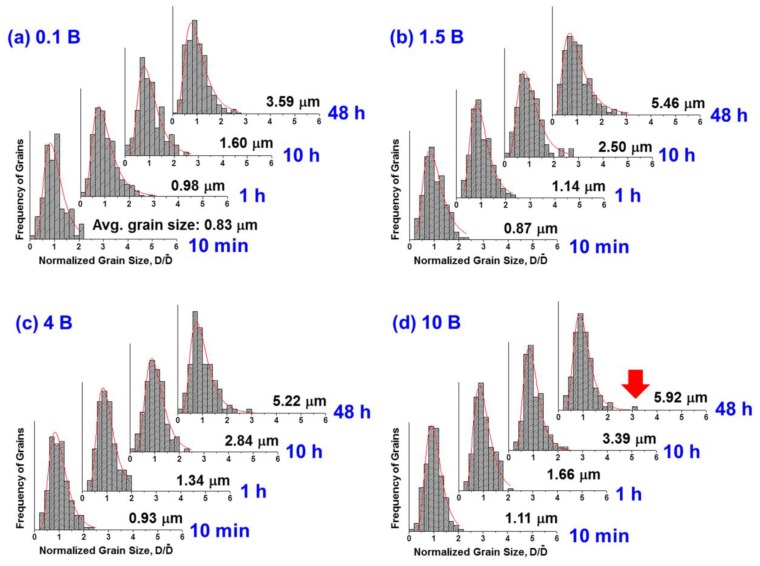
Measured normalized grain size distributions of (**a**) 0.1 mol%, (**b**) 1.5 mol%, (**c**) 4 mol%, and (**d**) 10 mol% Bi_2_O_3_-excess 0.95(Na_0.5_Bi_0.5_)TiO_3_-0.05BaTiO_3_ (NBT–5BT) samples sintered at 1200 °C for various sintering times.

Figure 2 and Figure 3 show microstructures and grain size distributions of the NBT–5BT samples sintered at 1200 °C for various sintering times and amounts of Bi_2_O_3_. The samples were prepared after polishing and chemical etching. In this study, grain growth behavior of undoped (0 mol% Bi_2_O_3_) NBT–5BT was not reported, but the average grain size decreased when 0.1 mol% Bi_2_O_3_ was added to NBT–5BT compared with our previous result [18]. However, as more Bi_2_O_3_ was added to NBT–5BT, the average grain size increased at each sintering time. Therefore, the phenomenon cannot be explained with only the Zener or solute drag effects because grain growth should be suppressed by both effects. Grain growth behaviors of almost all samples were similar to normal grain growth in which abnormal grains were not clearly observed in all cases in micrographs and grain size distributions. However, abnormal grains were detected in the grain size distribution of the 10 mol% Bi_2_O_3_-excess NBT–5BT sample sintered at 1200 °C for 48 h shown in Figure 3 (indicated by a red arrow). An abnormal grain can be defined as a grain whose size is over three times the average grain size [18].

As the amount of Bi_2_O_3_ increases, the corner shape of the grains becomes more faceted and a layer-structured secondary solid phase appears, as shown in Figure 2. The layer-structured secondary solid phase may be the secondary solid phase observed in the X-ray diffraction patterns shown in Figure 1. The grain shape change could change the grain growth behavior because the interface structure was changed [2,3,18,19,23,27,30,32,39]. Therefore, the grain growth behavior is affected by the change of the interface structure and the existence of the liquid phase. In our previous study, the grain growth of NBT–5BT was governed by the growth of facet interfaces and two-dimensional nucleation-controlled grain growth [18,19]. Grain growth behavior of undoped NBT–5BT with round-edged cubic grains is pseudo-normal grain growth as found in the previous study [18]. Usually, as the grain shape becomes more faceted, abnormal grain growth behavior could appear because of increasing step free energy [2,3,18,19,23,27,30,32,39]. However, the grain growth behavior does not change to abnormal grain growth as the amount of Bi_2_O_3_ increases in the present results, as is shown in Figure 2 and Figure 3.

**Figure 4:**
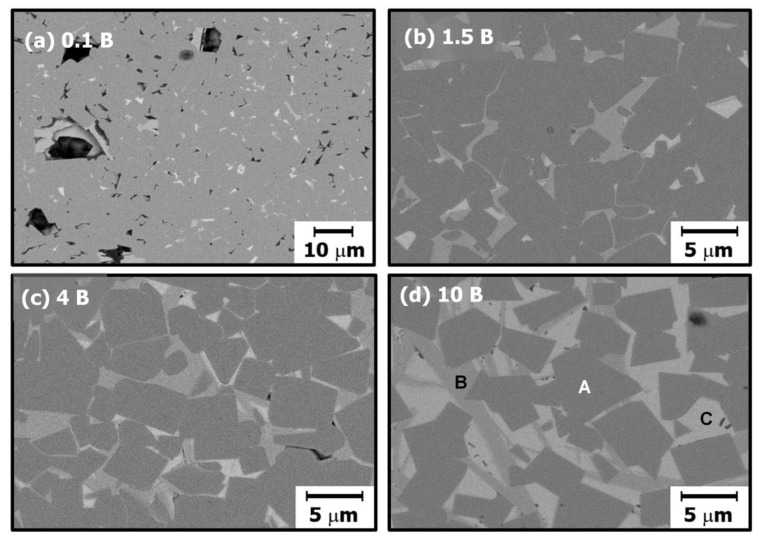
Backscattered SEM images (BSE mode) without chemical etching treatment of (**a**) 0.1 mol%, (**b**) 1.5 mol%, (**c**) 4 mol%, and (**d**) 10 mol% Bi_2_O_3_-excess 0.95Na_0.5_Bi_0.5_TiO_3_-0.05BaTiO_3_ (NBT–5BT) samples sintered at 1200 °C for 48 h.

To explain the grain growth behavior and microstructural change in more detail, the microstructures of samples were observed without chemical etching, because chemical etching can affect unstable phases such as a liquid phase. Figure 4 shows the backscattered SEM images of samples without chemical etching treatment. The samples were sintered at 1200 °C for 48 h. Table 1 presents the energy dispersive spectroscopy (EDS) analysis results of 10 mol% excess NBT–5BT sintered at 1200 °C for 48 h. In Figure 4d, the area marked (A) is an NBT–5BT grain, the dark grey area marked (B) is a layer-structured secondary solid phase, and the bright grey area marked (C) is a liquid phase. In the micrographs of samples without chemical etching, the liquid phase can be observed; however, the liquid phase cannot be observed in the micrographs of the chemically etched samples shown in Figure 3. The liquid phase was removed during the chemical etching treatment. According to the EDS results in Table 1, the amounts of Na and Bi were similar in area (A); however, sodium does not appear in areas (B) and (C). A relatively large amount of Bi was detected in area (C). Thus, area (A) could be NBT–5BT and the composition of the liquid phase in area (C) is close to Bi_2_O_3_. Usually, the liquid phase and its amount could affect grain growth behavior [2,13,23,24,26,40,41,42,43]. Material transport, that is, diffusion of atoms, is fast through the liquid phase [23,43]. Thus, the microstructure change is fast if a small amount of liquid phase is present. However, the diffusion rate of the material decreases as the amount of liquid phase increases because the diffusion distance is increased. Therefore, in a large amount of liquid phase, grain growth can be suppressed [2,26]. In addition, the liquid phase can change the interface structure, interfacial energy, and grain shape [2,26,32,40]. Finally, the grain growth behavior of NBT–5BT with the addition of Bi_2_O_3_ can be affected by the secondary solid phase, liquid phase, and critical driving force for growth via interfacial morphology and grain shape. In Figure 4, NBT–5BT grains change in shape from round-edged cubes to sharp-edged cubes and the fraction of liquid phase increases as the amount of Bi_2_O_3_ increases. Grain growth behavior of round-edged cubic NBT–5BT without Bi_2_O_3_ is similar to normal grain growth, but two-dimensional nucleation-controlled grain growth is governed by faceted interfaces [18]. In Figure 2 and Figure 4, the grain shape is becoming more faceted, that is, the round edges are sharpened with increasing amounts of Bi_2_O_3_. Faceting of the grain shape means that the step free energy increases and the critical driving force for growth also increases [3,14,17,19]. As explained in previous studies [2,3,19], the critical driving force (${\Delta g}_{c}$) is a value which determines whether or not appreciable grain growth occurs. It is expressed as ${\Delta g}_{c}=(\pi \sigma^{2}/kTh\;)$$\cdot {\left(\ln K\right)}^{-1}$, where σ and *h* are the step free energy and the height of the 2-D nucleus, and K is a constant including diffusion coefficient and number density of the nuclei. This equation indicates that the critical driving force is proportional to the square of the step free energy (σ) that is closely related to the grain shape [2,3]. When the critical driving force for growth increases, grain growth behavior can be changed to abnormal grain growth or stagnant growth [2,3,19]. In our previous study [18], obvious abnormal grains appear after 12 h of sintering time. However, obvious abnormal grains cannot be observed in this study, as shown in Figure 2 and Figure 4. Moreover, abnormal grains should appear easily because of the increased faceting of interfaces with the addition of Bi_2_O_3_, which increases the critical driving force accordingly. The reason why abnormal grain growth is suppressed with Bi_2_O_3_ is that the distance of diffusion increases as the amount of liquid phase increases with the increasing addition of Bi_2_O_3_, as shown in Figure 4. Although obvious abnormal grains cannot be observed, an abnormal grain can be found in the 10 mol% Bi_2_O_3_-excess NBT–5BT sample sintered at 1200 °C for 48 h, as shown in Figure 3. Average grain size increases without the solute drag effect as the amount of Bi_2_O_3_ increases, as shown in Figure 2. Most previous studies investigated the electrical properties in the NBT–5BT system; however, they focused on the effect of processing parameters such as synthesis methods [36,44,45,46], sintering temperature, morphotropic phase boundary (MPB) [47,48], and amounts of additive elements [37,49] on the microstructure and dielectric properties. A previous study [37] investigated the effect of adding Bi_2_O_3_ on microstructure and dielectric–temperature curves. However, that study did not focus on the reason for microstructural change with Bi_2_O_3_ addition, unlike the present study.

## 4. Conclusions

The grain growth behavior of NBT–5BT with excess Bi_2_O_3_ addition was investigated. The equilibrium shape of an undoped NBT–5BT grain is a round-edged cube. When Bi_2_O_3_ was added to round-edged cubic NBT–5BT, the average grain size decreased for 0.1 mol% Bi_2_O_3_-excess NBT–5BT compared with undoped NBT–5BT, but the average grain size then increased as the amount of Bi_2_O_3_ increased. The grain shape changed from round-edged cubic to sharp-edged cubic and the amount of liquid phase increased with the addition of Bi_2_O_3_. Abnormal grain growth behavior can be expected because the critical driving force for growth can increase with increasing amounts of Bi_2_O_3_. However, obvious abnormal grain growth did not appear in all of the NBT–5BT samples with excess Bi_2_O_3_. Another role of Bi_2_O_3_ is the formation of a liquid phase. The amount of liquid phase increased as the amount of Bi_2_O_3_ increased. Therefore, the abnormal grain growth could be suppressed by increasing the distance for the diffusion of atoms. Abnormal grains were detected in the grain size distribution of 10 mol% excess-Bi_2_O_3_ addition NBT–5BT, in which the largest grain was over three times the average grain size. This study allows us to conclude that the growth of Bi_2_O_3_-excess NBT–5BT grain is governed by the growth of facet planes via the two-dimensional nucleation-controlled grain growth mechanism.

## Figures and Tables

**Table 1:** Energy dispersive spectroscopy (EDS) analysis results with 10 mol% Bi_2_O_3_-excess 0.95(Na_0.5_Bi_0.5_)TiO_3_–0.05BaTiO_3_ (NBT–5BT) samples sintered at 1200 °C for 48 h shown in Figure 4d.

Area	Na	Bi	Ti	O
Grain (A)	11.94 at%	11.17 at%	26.52 at%	50.38 at%
2nd Phase (B)	–	27.78 at%	24.48 at%	47.74 at%
Liquid (C)	–	54.98 at%	04.21 at%	40.82 at%
